# The New Phytocomplex AL0042 Extracted from Red Orange By-Products Inhibits the Minimal Hepatic Encephalopathy in Mice Induced by Thioacetamide

**DOI:** 10.3390/biomedicines13030686

**Published:** 2025-03-11

**Authors:** Loredana Vesci, Giulia Martinelli, Yongqiang Liu, Luca Tagliavento, Mario Dell’Agli, Yunfei Wu, Sara Soldi, Valeria Sagheddu, Stefano Piazza, Enrico Sangiovanni, Francesco Meneguzzo

**Affiliations:** 1 Research and Development, Alfasigma S.p.A., 00071 Pomezia, Italy; loredana.vesci@gmail.com; 2 Department of Pharmacological and Biomolecular Sciences “Rodolfo Paoletti”, Università degli Studi di Milano, 20133 Milano, Italy; giulia.martinelli@unimi.it (G.M.); stefano.piazza@unimi.it (S.P.); enrico.sangiovanni@unimi.it (E.S.); 3 Department of Pharmacology, Discovery Services, BioDuro-Sundia, Shanghai 200131, China; john.liu@bioduro-sundia.com (Y.L.); yunfei.wu@bioduro-sundia.com (Y.W.); 4 HyRes S.r.l, 82100 Benevento, Italy; luca.tagliavento@hyres.it; 5 AAT Srl–Advanced Analytical Technologies, 29017 Fiorenzuola d’Arda, Italy; sara.soldi@aat-taa.eu (S.S.); valeria.sagheddu@aat-taa.eu (V.S.); 6 Institute of Bioeconomy, National Research Council of Italy, 50019 Florence, Italy

**Keywords:** minimal hepatic encephalopathy, inflammation, polyphenols, hydrodynamic cavitation, green extraction

## Abstract

**Background/Objectives**: Minimal hepatic encephalopathy (MHE) is a clinical condition characterized by neurological impairments, including brain inflammation, arising from the accumulation of toxic metabolites associated with liver dysfunction and leaky gut. This study investigated the pharmacological activity of a new phytocomplex extracted from red orange by-products (AL0042) using hydrodynamic cavitation and consisting of a mixture of pectin, polyphenols, and essential oils. **Methods**: Preliminary in vitro studies evaluated the impact on the epithelial integrity (TEER) of enterocytes challenged by a pro-inflammatory cocktail. The effect of AL0042 was then evaluated in a model of thioacetamide (TAA)-treated mice that mimics MHE. A group of 8–10-week-old male C57BL/6 mice was intraperitoneally injected with TAA to establish the MHE model. The intervention group received TAA along with AL0042 (20 mg/kg, administered orally once daily for 7 days). At the end of the treatment, the rotarod test was conducted to evaluate motor ability, along with the evaluation of blood biochemical, liver, and brain parameters. **Results**: In vitro, AL0042 (250 μg/mL) partially recovered the TEER values, although anti-inflammatory mechanisms played a negligible role. In vivo, compared with the control group, the test group showed significant behavioral differences, together with alterations in plasma ammonia, serum TNF-α, ALT, AST, corticosterone levels, and SOD activity. Moreover, histological data confirmed the anti-inflammatory effect at liver and brain level. **Conclusions**: AL0042 treatment revealed a significant therapeutic effect on the TAA-induced MHE mouse model, curbing oxidative stress and peripheral and central inflammation, thus suggesting that its pharmacological activity deserves to be further investigated in clinical studies.

## 1. Introduction

The neuropsychiatric syndrome of minimal hepatic encephalopathy (MHE) because of cirrhosis or acute and chronic liver failure (ACLF) may become evident with different symptoms. These are personality change, confusion, forgetfulness, reduced attention, lethargy, cerebral oedema, and other complications progressing to coma [1,2].

In some studies, altered neurotransmitter homeostasis has been identified as a contributing factor to the neurological manifestations of MHE, in addition to excessive production of ammonia caused by liver damage, alterations in intestinal microbiota, and inflammatory processes [3,4]. Indeed, liver and intestinal failure induced hyperammonemia leading to systemic inflammation [5], and neuroinflammation, as suggested by the activation of microglia in MHE patients [6], and in animal models [7,8]. The correlation between gut microbiota dysbiosis and the pathogenesis of MHE was documented by Luo and colleagues, in which the efficacy and safety of microbiome therapies for MHE were also demonstrated [9]. In more detail, the disruption in neurotransmission systems leads to neuronal disinhibition. Emerging studies indicate that when the lymphatic system, which plays a role in clearing accumulated brain toxins, is compromised, it may contribute to the onset of MHE. Under such conditions, astroglia have been shown to generate tumor necrosis factor (TNF-α), triggering glutamate release and microglia activation. This activation often results in the proliferation of microglia and the secretion of pro-inflammatory cytokines such as TNF-α, interleukin-1 (IL-1), and interleukin-6 (IL-6). Both animal and human studies suggest that elevated ammonia levels alone are insufficient to cause MHE unless they are accompanied by a systemic inflammatory response. Consequently, it is widely accepted that sepsis-induced alterations in nitrogen metabolism, along with the release of pro-inflammatory mediators, may be key factors in the development of MHE in patients with cirrhosis [10].

To study the effect of new products, the hepatotoxic agent thioacetamide (TAA) was administered intraperitoneally for a short time in the MHE animal model, resulting in a reproducible model that effectively mimicked the liver injury observed in ACLF and MHE patients. TAA metabolites induce oxidative stress and liver injury, raise the aspartate aminotransferase (AST) and alanine aminotransferase (ALT) levels, and trigger both systemic and neuronal inflammation, ultimately leading to hyperammonemia and cerebral damage [11].

The application of hydrodynamic cavitation (HC), an emerging, energy-efficient, and green extraction technique [12], to citrus fruit by-products (the peel, seeds, and part of the pulp, resulting from juice squeezing) yielded pharmacologically active natural products. The pilot-scale processing of orange by-products (42 kg fresh weight) with 120 L of water allowed the extraction of a phytocomplex composed of pectin with a very low degree of esterification (DE, percent of methyl-esterified carboxyl groups) of 17.05%, with hesperidin, naringin, other flavonoids, and volatile compounds dominated by limonene, adsorbed onto the surface of pectin [13,14]. The same HC process has also been successfully applied to other citrus waste streams, such as lemon and grapefruit [15], and a grapefruit extract obtained by HC was later used for an in vivo study concerning cardiac protection [16]. Besides efficiency and the ability to generate stable conjugated phytocomplexes, the straightforward scalability of HC processes represented a key advantage from the perspective of industrialization.

Red orange, also known as blood orange (*Citrus* × *sinensis* L.), is a valuable variety of sweet orange renowned for its rich content of health-promoting bioactive compounds, including flavonoids (with hesperidin dominating in the juice and peel), limonoids (such as the limonoid glucosides found in the juice and pulp and limonoid aglycones present in the seeds), and anthocyanins (for example, malvidin 3-*O*-glucoside). Red orange peels, as the most abundant component of by-products (peels, pulp, and seeds), were previously reported to possess a total number of bioactive compounds around 46.7 g/kg, 98.7% of which were flavonoids, while the juice contained a similar number of bioactive compounds but far less flavonoids (8.6 g/kg) [17]. Hesperidin, accounting for about 86% of the total flavonoids in peels, was shown to cause anti-inflammatory activity, including toward liver inflammation, which is relevant to the development of MHE [18,19]. Thus, red orange by-products appear to be the most promising fraction of the fruit in relation to the purposes of this study.

In this study, we report for the first time the ability of the new phytocomplex AL0042, derived from HC-based integral extracts of red orange by-products, to counteract MHE, lowering inflammation, oxidative stress, and cerebral anomalies in a murine model of MHE. The in vitro studies also demonstrated that the extract exerts anti-inflammatory and epithelial protective effects on enterocytes, which give the product a prebiotic value deserving further studies.

## 2. Materials and Methods

### 2.1. Extraction of Red Orange By-Products

Red orange by-products were obtained from the squeezing of whole fruits of the Tarocco variety, grown according to the specifications set by the Consorzio Arancia Rossa di Sicilia IGP, the agency aimed at regulating and protecting red orange cultivation in Sicily (https://www.tutelaaranciarossa.it/, accessed on 27 January 2025). The extraction runs were performed using a non-commercial HC pilot plant developed by the Institute of Bioeconomy, National Research Council of Italy, 200 L in total volume, and water as the only solvent, according to the procedure described in a previous study and using the same equipment [13]. Two extraction runs were performed, the first aimed at optimizing the production of the dry extract, the second at producing sufficient dry extract for the in vivo trial. Together, such runs were also useful to assess the degree of standardization of the extract. The runs were performed on 11 March 2022 (“ALE1”) and 20 January 2023 (“ALE2”), within 24 h of receiving the respective frozen batches of red orange by-products, in turn resulting from fruits harvested within a week before. The weight and physical properties of the materials used in the extractions were identical: 48.5 kg of fresh biomass (red orange by-products) with a moisture content of 75%, mixed with 121 L of tap water, so that the dry biomass to water ratio was 1:10. ALE1 and ALE2 started at the temperatures of 10.5 and 7.0 °C, respectively, which was the only difference between the tests, and both were stopped at the temperature of 47 °C, after 72 and 85 min, with specific energy consumption of 0.67 and 0.78 kWh per kg of processed dry biomass, respectively. The aqueous extracts collected at the end of the processes (temperature of 47 °C) were filtered with a 50 µm sieve (stainless steel mesh) and stored in sterile bottles at −20 °C until use.

To ensure reproducibility, further details of the HC-based extraction process are provided. The centrifugal pump afforded a constant discharge of 53.8 m^3^/h; thus, the cavitation passes were 380 and 450 in ALE 1 and ALE2, respectively.

With static HC reactors, such as the Venturi-shaped one used in this study, cavitation regimes can be represented using a dimensionless parameter known as the cavitation number (σ), based on Bernoulli’s law and expressed in Equation (1):

(1) $$\sigma =\frac{p_{2}-p_{sat}}{0.5\rho u^{2}},$$

where *p*_2_ is the recovery pressure downstream the throat (assumed at the level of the atmospheric pressure), *p_sat_* is the temperature-dependent saturation vapor pressure of the liquid; *ρ* is the temperature-dependent liquid density; and *u* is the flow velocity through the reactor throat, which depends only on pump discharge. The intensity of cavitation events, namely the phenomena related to the collapse of cavitation bubbles, such as extreme temperatures, pressure shockwaves, hydraulic jets and the generation of free radicals, increases with decreasing cavitation number until sudden damping due to the onset of chocked cavitation. In distilled water, the range 0.1 < σ < 1 corresponds to developed cavitation [20]. The geometrical features of the HC reactor and the quantitative assessment method of σ in the reactor for any process temperature were previously published [13].

Cavitation usually occurs as well around the impeller of a centrifugal pump and can be described by the same cavitation number as in Equation (1) [21], whose computation is based on the impeller diameter (174 mm) and rotational speed (2900 rpm), resulting in the velocity of the mixture at the impeller tip (*u* in Equation (1)) of 26.4 m/s, again assuming *p*_2_ at the level of the atmospheric pressure.

### 2.2. Preparation and Analysis of AL0042

AL0042 was prepared by spray drying the integral, non-purified aqueous extracts of red orange by-products, using the Spray dryer model APT-2.0 (APTSOL, Novara, Italy). The choice of spray drying as the isolation method was based on its straightforward scalability and widespread industrial use. The particle size distribution (PSD) analysis was performed using Bettersize 2600 dry (3P instruments, Odelzhausen, Germany). The thermogravimetric analysis was performed using thermobalance RADAWAG MA 50/1.R.WH (Radom, Poland).

The optimization of the spray drying was performed with the aqueous extract collected from ALE1, based on the maximization of the recovery of the total dissolved solids, varying the content of red orange by-products, along with the excipient’s maltodextrin (DE 18-20, Farmalabor, Assago, Milan, Italy) and hydroxypropyl methylcellulose (HPMC) (Metochel e5, Colorcon, Harleysville, PA, USA). AL0042 was obtained from the aqueous extract of ALE2 using the optimized spray drying the process parameters.

These specific excipients were chosen because they are the most common in supplements, ensuring a formation of the powder according to the needs and maintaining solubility. Maltodextrin is perfectly soluble; thus, it does not affect the product; it reduces the hygroscopicity of the powder and allows a more uniform formation, combined with the increase in solubility of the final compound. HPMC, which is a little less soluble compared to maltodextrin, helps the formation of the powder when the water particle evaporates in the spray drying tower. These excipients have been used to improve the spray drying process yields, improve drying, and reduce the final residual humidity, with an advantage in conservation. However, other excipients that do not increase the glycemic index might be evaluated.

A comprehensive analysis of the proximate and chemical composition of the final product was performed in the form of spray-dried powder obtained from the aqueous extracts of ALE1 and ALE2, the latter providing AL0042. The analyses were carried out by CHELAB S.r.l. Sole Partner Company (ACCREDIA LAB N° 0051 L, Milano Rho, Italy), subjected to the direction and coordination of Merieux NutriSciences Corporation (Resana, Italy), following internal methods based on official methods. All measurements were made in duplicate. Table 1 lists the analyzed quantities and the respective methods.

### 2.3. Total Phenol Content Assay

The total phenol content was determined by the Folin–Ciocâlteu’s method, as previously described [29]. In brief, the spray-dried extract was dissolved in water, then a 2 N Folin–Ciocâlteu solution (Merck Life Science, Milan, Italy) and a Na_2_CO_3_ solution (20% *w*/*v*) were added. Following a 30 min incubation period at 37 °C, the absorbance of the samples was measured with a spectrophotometer (Jasco V630, JASCO International Co. Ltd., Tokyo, Japan) at 765 nm, with the results expressed as mg of gallic acid equivalents per g of extract using a calibration curve of gallic acid as a reference compound.

### 2.4. Oxygen Radical Absorbance Capacity

The oxygen radical absorbance capacity (ORAC) of the spray-dried extract was determined as previously described [29]. In brief, the spray-dried extract was transferred into a black 96-well plate, followed by the addition of a fluorescein solution (dissolved at 70 nM in a phosphate buffer at pH 7.4). Subsequently, 2,2′-azobis(2-methylpropionamidine) dihydrochloride (AAPH) (40 mM) was added to each well, resulting in the generation of peroxyl radicals. The fluorescein and AAPH were supplied by Merck (Merck Life Science, Milan, Italy). The fluorescence (excitation/emission: 484/528 nm), following the shaking of the plate at 37 °C every 2 min for 60 min (Victor^TM^ X3, Perkin Elmer, Waltham, MA, USA), was read, and the area under the curve (AUC) of the spray-dried extract was calculated, with the results expressed as mmol Trolox equivalent per g of extract using a curve of Trolox (0–120 μM) (Merck Life Science, Milan, Italy) as a reference inhibitor.

### 2.5. In Vitro Investigations

#### 2.5.1. Cell Culture and Treatments

The human epithelial cells, derived from colorectal adenocarcinoma (Caco-2, clone HB237, ATCC, Manassas, VA, USA), were maintained in a 75 cm^2^ flask under a humidified atmosphere with 5% CO_2_ at 37 °C with high-glucose DMEM (Merck Life Science, Milan, Italy) supplemented as follows: 1% penicillin/streptomycin (10,000 U/mL), 2% L-glutamine (200 mM), 1% non-essential amino acids, 1 mM sodium pyruvate (Gibco^TM^, Thermo Fisher Scientific, Rodano, MI, Italy), and 10% fetal bovine serum (FBS, Euroclone S.p.a., Pero, MI, Italy). Every 2 to 3 days, the cells were detached using Trypsin-EDTA 0.25% (Gibco^TM^, Thermo Fisher Scientific, Rodano, MI, Italy), avoiding the confluency. The number of cells was counted and transferred into a new flask to maintain the cell line or seeded (3 × 10^5^ cells) on 12-well Nunc Cell Culture Inserts (Nunc^TM^, Thermo Fisher Scientific, Rodano, MI, Italy), where the colonocyte cells were cultivated after confluency and differentiated to enterocytes cells in 17–21 days. During this period, every 2 days, the apical compartment received FBS-free medium, while the basolateral compartment was provided with a complete medium. On the day of the treatment, the cells were treated with a pro-inflammatory cocktail (TNF-α 50 ng/mL, INF-γ 50 ng/mL, IL-1β 25 ng/mL, and LPS 1 mg/mL) for a period of 24 h, along with the spray-dried extract at 250 μg/mL or the pure molecule hesperidin at 30 μM (Phytolab GmbH & Co. KG, Vestenbergsgreuth, Germany), before the assessment of cytotoxicity, inflammation, and permeability. The pro-inflammatory cocktail was selected to reproduce an inflammatory response typically mediated by leukocytes (TNF-α, IL-1β, and IFN-γ), which is associated with the translocation of bacterial structures (i.e., LPS) during leaky gut syndrome. Cytokines were also selected based on previous studies regarding the responsiveness of Caco-2 to inflammatory mediators [30].

The extract and hesperidin were dissolved by a DMSO:H_2_O (1:1) mixture, aliquoted, and stored at −20 °C before experiments. The treatments were conducted using FBS-free medium, and the cells were maintained at 37 °C with 5% CO_2_ in humidified atmosphere. All disposable materials were from Primo^®^ or Falcon^®^ (Euroclone S.p.a., Pero, MI, Italy; Corning Life Sciences, Amsterdam, The Netherlands).

#### 2.5.2. Cell Viability (MTT Test)

The viability of the Caco-2 cells was determined using the 3-(4,5-dimethyl-2-thiazolyl)-2,5-diphenyl-2H-tetrazolium bromide (MTT) solution, as previously described [31]. In brief, MTT was dissolved in PBS 1× and added to the cells at the end of the 24 h treatments with spray-dried extract and hesperidin. Following a 45 min incubation period at 37 °C, the cells were lysed with an isopropanol:DMSO (90:10 *v*/*v*) solution. The absorbance was measured at 550 nm using a spectrophotometer (Victor^TM^ X3, Perkin Elmer, Waltham, MA, USA), and the resulting value was directly correlated with cell viability.

#### 2.5.3. Measurement of Cell Epithelial Integrity

Following a 24 h treatment of Caco-2 cells, in the form of enterocytes, with an inflammatory cocktail and extract or hesperidin simultaneously added to the apical side of the well, the epithelial integrity was evaluated by measuring the transepithelial electrical resistance (TEER) opposed by the cells’ monolayer. The TEER values (Ω) were determined using the EVOM3 device (WPI, Sarasota, FL, USA) at the outset of each experiment (t0), with a cut-off value of 400 Ω, and at the conclusion of each experiment (t24). The variation in TEER values (∆Ω) was subsequently calculated (∆Ω = Ωt24h − Ωt0), and the data were expressed as the mean of ∆Ω ± SEM of at least four independent experiments. The data were analyzed using the GraphPad Prism 9.0 software (GraphPad Software Inc., San Diego, CA, USA), with an unpaired ANOVA test and Bonferroni post-hoc analysis. A *p*-value of less than 0.05 was considered statistically significant. Sodium butyrate (2 mM) was used as a reference trophic factor for the gut barrier.

#### 2.5.4. Measurement of Inflammatory Parameters

Following a 24 h treatment of Caco-2 cells, in form of enterocytes, the anti-inflammatory activity of the spray-dried extract and hesperidin, which were simultaneously added to the apical side of the well with the inflammatory cocktail, was evaluated on 84 inflammatory genes using an RT-PCR array (RT^2^ Profiler^TM^ PCR Array Human Inflammatory Cytokines & Receptors, QUIAGEN S.r.l., Milan, Italy), as previously described [32]. The cells were lysed using QIAZOL Lysis Reagent (QUIAGEN S.r.l., Milan, Italy), and the RNA isolation was performed using the NucleoSpin^TM^ RNA Mini purchased from Machery-Nagel^TM^ (Carlo Erba Reagents S.r.l., Milan, Italy). An aliquot of cDNA, corresponding to 400 ng of total RNA, was then mixed with the SYBR Green Master Mix RT^2^ reagent (QIAGEN S.r.l., Milan, Italy) and loaded into the 384-well array. The RT-PCR was conducted using the CFX384^TM^ Real-Time PCR Detection System, which was coupled to a C1000^TM^ Thermal Cycler (Bio-Rad Laboratories S.r.l., Segrate, Italy). The threshold cycle value for each gene (C_T_) was automatically provided by the management software CFX Manager^TM^ 2.1 (Bio-Rad Laboratories S.r.l., Segrate, Italy), depending on the amplification curves. The cycle threshold (C_T_) cut-off was established at 35, and the housekeeping gene RPLP0 was employed for data normalization. The data analysis web portal employs the delta C_T_ method to calculate fold change/regulation, whereby Fold Change (FC) is determined as FC = 2^−∆∆Ct^ to quantify alterations in gene expression between the treated and control groups. The analysis of the two experiments was conducted using the web portal at GeneGlobe (QIAGEN Sciences, Germantown, MD, USA).

### 2.6. In Vivo Model to Induce Hepatic Encephalopathy

Male C57Bl/6J mice (8 weeks of age, Vital River Laboratory Animal Care Co., Ltd., Shanghai, China) were housed in stainless steel cages at 22 °C ± 1 °C with a 12:12 h light–dark cycle with food and water ad libitum. The number of animals for each group was calculated with the method Gpower3.1.9.4 [33]. For hypothesis-testing studies, the primary outcome measure used to determine the sample size was the behavioural parameter (latency fall) based on other previous experiments. The criteria for including animals were established before starting the experiments, based on a body weight of the animals of at least 18–20 g and their health status. A study protocol describing the key design features and analysis plan was prepared before the study and kept in our archives.

All animals were labeled with ear tags and, after 7 days’ acclimation, randomized into four groups by the random number table method with 10 mice for each, based on body weight (*n* = 40). The mice were divided into control and MHE groups. In the MHE groups, thioacetamide (TAA, Sigma-Aldrich, Catalog Number: 62-55-5, St. Louis, MO, USA) solubilized in saline was administered intraperitoneally at 100 mg/kg on days 1 and 2 and then at 50 mg/kg on days 3 through 7. A group of sick mice received AL0042 at 20 mg/10 mL/kg dissolved in PBS by oral route daily for a week, starting 2 h after the first dose of TAA. This dose of AL0042 was chosen based on a previous tolerability and efficacy evaluation at higher doses (160, 80, 40, 20 mg/kg, po, once daily) in a TNBS murine colitis model. All the in vivo data obtained were the means ± SE 10 mice per group. No lethality induced by AL0042 was observed during the experiment.

### 2.7. Ethics Statement

All the animal procedures and care were carried out according to national and international laws and policies. The normal, age-matched mice (C57Bl/6 from Charles River, China) were not treated with a vehicle or drug and were used as controls. Rodent care and use were conducted in accordance with all applicable assessment and accreditation of laboratory animal care (AAALAC) regulations and guidelines. The experimental protocol was reviewed and approved by the Institutional Animal Care and Use Committee (IACUC) of BioDuro Biologics Co., Ltd. (Shanghai, China), on 30 January 2023; approval No.—BD-202301017-1.

### 2.8. Body Weight and Behavior Test

The animals were subjected to daily body weight measurements, and clinical observations were registered. An accelerating rotarod motor learning test was performed, according to previously established methods [34], with a mouse rotarod fatigue tester (Cat: ZS-RDM-XS, Beijing Zhongshi Dichuang Technology Development Co., Ltd., Beijing, China).

In summary, the mouse was positioned on a motorized rod (3 cm in diameter, 5.7 cm in width, and 16 cm in height) for preadaptation. This was followed by an acceleration program that increased the rotation speed from 2 rpm to 80 rpm over a 5 min period. The time until the mouse fell (latency to fall) was recorded. To reduce variability in baseline performance, an initial test session was conducted prior to TAA administration (day 0). Three consecutive daily training sessions were performed from day 4 to day 6. Within each session, three trials were performed, and the average performance was recorded. The behavior test was carried out on one mouse/group from group 1 to group 4, four mice as one round, for a total of ten rounds.

### 2.9. Biochemical Parameters Analysis

On day 8, before anaesthesia, 20 μL of peripheral blood from the tail punctures of the mice were used with the blood ammonia Meter (Arkray, PocketChem BA PA-4140, Nagasaki, Japan) to detect the content of ammonia. After plasma ammonia, all animals were euthanized with CO_2_. Blood was collected through cardiac blood sampling, and the serum was separated and stored at 80 °C for the detection of ALT, AST, tumor necrosis factor-alpha (TNF-α), superoxide dismutase (SOD), and corticosterone contents. Biochemical parameters were measured with ELISA kits: ALT (IFCC, Wako, catalog number 998-62491, Neuss, Deutschland), AST (IFCC, Wako, catalog number 998-61891, Neuss, Deutschland), TNF-α (Invitrogen, catalog number 88-7324, Waltham, MA, USA), SOD activity (Merck, catalog number 19160, Rahway, NJ, USA), and corticosterone levels (ELISA Kit: Abcam, Catalog Number: ab108821, Cambridge, UK) by thermouse ELISA kit (Uscn life Science Inc., Huston, TX, USA) according to the manual instructions. Three replicates were performed for each sample. The scientists who conducted the assays were blind to the grouping information.

### 2.10. Liver Histological Evaluations

The tissue samples were preserved in 4% paraformaldehyde for 48 h, after which they were embedded in paraffin using a Leica EG1150H instrument (Leica Instrument Shanghai Ltd., Shanghai, China) and sliced into 4-mm sections with a Leica RM2235 microtome (Leica Instrument Shanghai Ltd., Shanghai, China). The slices were then dewaxed and stained with hematoxylin-eosin (H&E; ServiceBio Co., Ltd., Wuhan, China). Finally, the stained slices were examined and photographed under a light microscope (Nikon Eclipse CI-L, Tokyo, Japan) using a 20× objective lens. The standard of pathological score was defined according to Table 2 to evaluate the vacuolization, inflammation, and focal necrosis rate [35]. The pathologist who read the slices was blind to the grouping information.

### 2.11. Immunohistochemical Analysis in Brain

The mice were perfused transcardially with phosphate-buffered saline (PBS). The brains were separated, and the primary motor cortex was collected and soaked in 4% paraformaldehyde for 48 h. Then, a 30% sucrose solution was used for dehydration treatment. The brain motor cortex was selected for immunohistochemical analysis; first, they were embedded with an OTC reagent, and sections were cut at 8 μm. The frozen tissue sections were sealed with 10% formalin and washed. The primary antibodies anti-glial fibrillary acid protein (GFAP) (Cell Signaling Technology cat no 80788, Danvers, MA, USA), macrophage/phagocytic activation (CD68) (Cell Signaling Technology cat no E307V, Danvers, MA, USA), and ionized calcium-binding adapter molecule-1 (IBA-1) (Abcam, cat no 289874, Cambridge, UK) were used. The dilution of the primary antibody was CD68 and IBA-1, 1:400 and 1:100 ratio, respectively; GFAP, 1:400 ratio. The primary antibody was incubated overnight at 4 °C. The secondary antibody F(ab’)-goat anti-rabbit HRP diluted 1:1000 (Abcam no ab6013) was incubated 60 min at room temperature. TUNEL kit was Abcam, no ab66110 was also used. NeuN primary antibody was Abcam no Ab177487 diluted 1:300 with donkey serum. Fluorescent secondary antibodies were diluted 1:500 with PBS and added to the slides. The samples were examined under a fluorescence microscope (Olympus, BX53, Tokyo, Japan). All measurements were taken using the same magnification of image (40×), and the quantification of cell numbers was performed by Image J 4. The scientist who analyzed the slices was blinded to the grouping information.

### 2.12. Statistical Analysis

All data shown are presented as mean value ± S.E.M. Statistical analysis was performed by one-way or two-way ANOVA (*n* = 10 animals in each group). Bonferroni post hoc analysis was performed for in vitro data, while a Shapiro–Wilk normality test was performed on in vivo data. Differences among groups were considered significant at values of *p* < 0.05. Analyses were performed using GraphPad Prism 9 (GraphPad Software, San Diego, CA, USA).

## 3. Results

### 3.1. Quantitative Analysis of AL0042 in Different Batches

**Figure 1:**
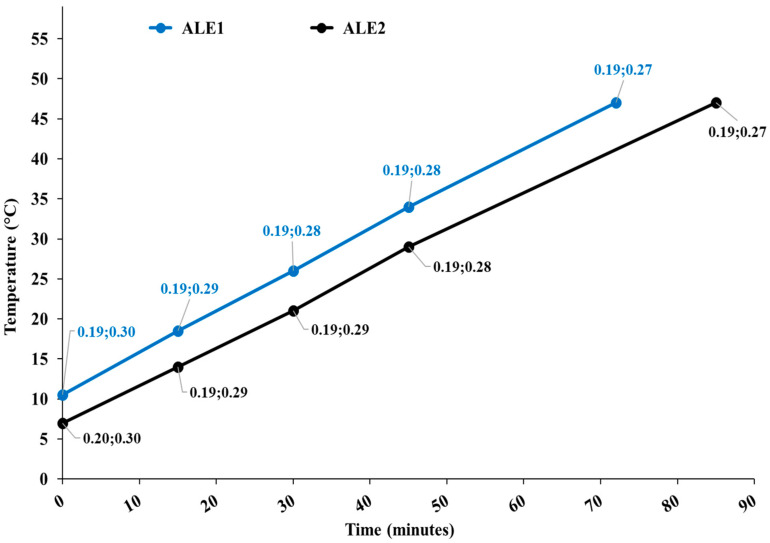
Temperature diagram as a function of the process time for extraction runs ALE1 and ALE2. Data tags show the levels of the cavitation number in the static HC reactor and at the pump impeller.

Figure 1 shows the temperature diagram as a function of the process time for both extractions, along with the cavitation number in the static HC reactor and at the pump impeller.

Apparently, processes were nearly identical for ALE1 and ALE2, and developed cavitation regimes occurred in both cavitation zones, with more intense cavitation (lower cavitation number) in the static reactor.

Table 3 lists the components and PSD of the product, in the form of spray-dried powder, including AL0042 derived from ALE2, along with the extraction yield.

All the characteristics and properties of the obtained dry powder under the same spray-drying conditions were reproducible across the two extractions. In particular, 90% and 50% of the particles had a size less than about 11 μm and 6 μm, respectively, with indistinguishable span in the 10% to 90% size distribution range. The micronized powder was also free-flowing.

Table 4 summarizes the proximate and chemical composition of AL0042 prepared from dry extracts of tests ALE1 and ALE2. Only quantities observed above the detection level in at least one of the extracts are represented. Since the end products from ALE1 and ALE2 (AL0042) were composed by red orange by-product extract in the amount of 74.97 ± 0.13% and 74.86 ± 0.04%, respectively, the contents of the listed quantities in the original dry extract of the red orange by-products are about 25% greater than the indicated levels.

AL0042 prepared from ALE2 extract was characterized by lower levels of total sugars, likely due to the collection of orange by-products earlier in the harvest season, and higher levels of pectin, dietary fiber, and slightly higher levels of the flavonoid hesperidin. Among aromatic volatiles, the share of limonene was higher in ALE2. Carbohydrates accounted for as much as about 78% of the composition of AL0042 from ALE2. The net of total sugars, the above differences would considerably reduce, with the content of hesperidin in ALE1 and ALE2 becoming practically indistinguishable. This evidence points to a good degree of standardization for extracts obtained from red orange by-products, resulting from fruits harvested in different years and different times of the harvest season, under the same extraction conditions described in Section 2.1. However, further investigation is recommended to confirm and more firmly establish the actual degree of standardization.

### 3.2. Evaluation of the In Vitro Anti-Inflammatory and Permeability Activity of Spray-Dried Extract (AL0042)

To provide a brief characterization of the spray-dried extract (AL0042), the antioxidant capacity was evaluated using the ORAC test, resulting in a mean value of 22,043.78 ± 1014.12 μmol Trolox _eq._/g (mean of at least three experiments ± S.D.). Furthermore, the extract was analyzed for the total phenolic content (TPC), which exhibited a value of 81.42 ± 3.15 mg gallic acid _eq._/g (mean of at least three experiments ± S.D.). Lastly, the amount of hesperidin, the primary flavonoid constituent of citrus species [17], was determined through MS spectrometry, resulting in 9.2% in the spray-dried extract. These preliminary analyses were useful in determining the set of further experiments and revealed that the spray-dried extract, due to its antioxidant activity and phenolic content, could be of interest with relation to anti-inflammatory and permeability activities. A single dose of the spray-dried extract (250 μg/mL) and the corresponding amount of hesperidin (20 μg/mL, equivalent to 30 μM) were selected for analysis based on either cytotoxicity or preliminary anti-inflammatory assays.

**Figure 2:**
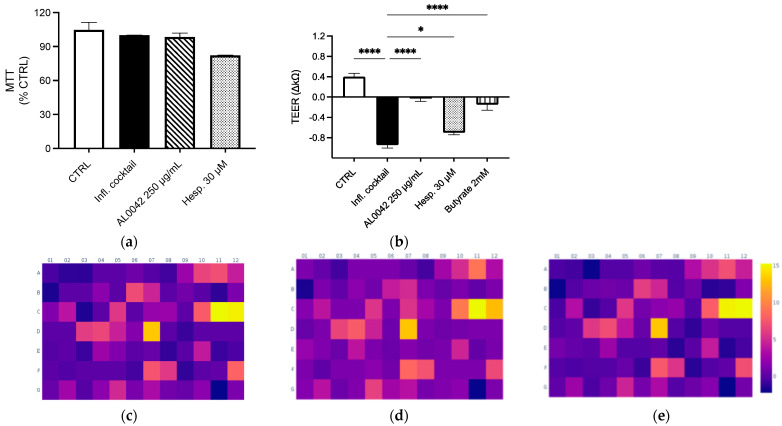
Evaluation of the effect of spray-dried extract and hesperidin on (**a**) Cytotoxicity; (**b**) Epithelial integrity. These measurements were performed in triplicate, and the error bars represent the standard error of the mean. Upregulation of genes after treatment with the inflammatory cocktail: (**c**) Control; (**d**) AL0042; (**e**) Hesperidin. Caco-2 cells, as enterocytes, were exposed to inflammatory cocktail (TNF-α 50 ng/mL, INF-γ 50 ng/mL, IL-1β 25 ng/mL, and LPS 1 mg/mL) and spray-dried extract at 250 μg/mL (AL0042) or pure molecule hesperidin at 30 μM (Hesp.) for 24 h. The cell viability was assessed using MTT test (**a**), where data were expressed as % of viability in respect to the stimulated condition. The epithelial integrity was assessed with the measurement of TEER (**b**), where data were expressed as variation in TEER values (∆Ω = Ωt24 h − Ωt0), * *p* < 0.05, **** *p* < 0.0001 vs. inflammatory cocktail. The impact on selected inflammatory genes was assessed with RT-PCR (**c**–**e**). The modulation (fold change) of the inflammatory cocktail on inflammatory genes was represented in the heatmap (**c**), while the impact (fold change) of the spray-dried extract and hesperidin on inflammatory genes was represented in the heatmaps (**d** and **e**, respectively), all relative to the unstimulated control.

Both the extract and the pure compound did not show any cytotoxicity in Caco-2 cells differentiated in Nunc Cell Culture Inserts for 17–21 days (Figure 2a). Mild inhibitory effects on NF-κB activation and IL-8 release (less than 20%) were demonstrated in undifferentiated Caco-2 after treatment, with the higher concentration belonging to AL0042 (250 μg/mL). The spray-dried extract demonstrated a significant effect on the epithelial integrity of enterocytes, comparable to that of the reference compound butyrate, in response to the inflammatory cocktail that caused epithelial destruction at the junction of cells. However, the pure molecule hesperidin exhibited a diminished activity, which does not explain the observed effect of the extract (Figure 2b).

In the same context, we investigated whether the extract and molecule exert an anti-inflammatory effect on a wider range of inflammatory genes following the stimulation of an inflammatory cocktail on enterocytes cells. The array, comprising 84 genes involved in inflammation (listed in Table 5), demonstrated the genes most up-regulated by the inflammatory cocktail, including those belonging to the CXC chemokine class, such as *CXCL-1*, *CXCL-2*, *CXCL-8*, *CXCL-9*, *CXCL-10*, and *CXCL-11*; genes belonging to the CC chemokines, such as *CCL-2*, *CCL-20*, and *CCL-22*; and TNF-related genes, such as *LTB*, *TNFSF*, and *TNF* itself (Figure 2c). The dried-spray extract (250 μg/mL) did not substantially alter the inflammatory profile induced by the cytokine cocktail; however, it demonstrated a modest inhibitory effect on the transcription of two genes known to be sensitive to IFN-γ stimulation, namely *CXCL-10* and *CXCL-11*, in addition to *CCR5* and *IL16* (Figure 2d). A similar outcome was observed in the treatment with hesperidin (30 μM) (Figure 2e). In line with these data, no inhibitory effect was observed at the protein level after measuring CXCL-10 release by ELISA assay on the culture media.

### 3.3. Effect of AL0042 on Body Weight and Behaviour of Mice

**Figure 3:**
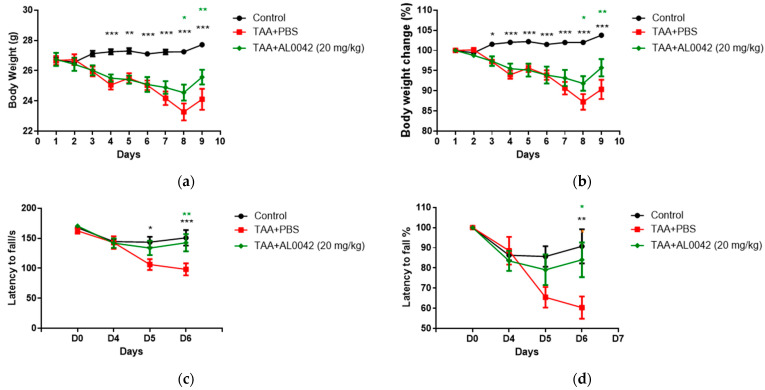
Body weight and abnormal behavior in control, TAA-induced, and TAA + AL0042-treated mice: (**a**) Time change of body weight with time; (**b**) Percentage time change of body weight with time; (**c**) Time change of latency to fall with time; (**d**) Percentage time change of latency to fall with time. C57BL/6 male mice were intraperitoneally treated daily with thioacetamide for a week. From day 1 to 7, AL0042 was administered by oral route at 20 mg/kg (*n* = 10). Body weight was registered daily, and the results were expressed as the mean of measurements performed in triplicate ± SE, * *p* < 0.05, ** *p* < 0.01, *** *p* < 0.001, were reported in green (AL0042 versus TAA-PBS) or black (control versus TAA-PBS), vs. TAA-PBS group, two-way ANOVA.

AL0042 administration macroscopically attenuated the body weight reduction evident in the TAA-treated mice (Figure 3a,b). It was evident that TAA decreased body weight compared with the untreated normal group starting from the second day. Compared with the control group, the sick group showed a significant depression in body weight from day 4 to 8 (*p* < 0.01, *p* < 0.001), and AL0042 treatment (20 mg/kg, po daily) revealed a significant improvement effect in percentage of weight loss changes on days 7 to 8, with respect to the TAA–PBS group (*p* < 0.05, *p* < 0.01, *p* < 0.001) (Figure 3a,b).

With regards to the behavior of the mice, measured using the Rotarod test, compared with the control group, the latency to fall of the model group showed a significant lowering at D5 and D6 (Figure 3c,d), highlighting the decline in exercise capacity in model animals. Compared with the model group, AL0042 treatment (20 mg/kg) displayed a significant improvement effect in exercise capacity at D6 (*p* < 0.05).

### 3.4. Effect of AL0042 on the Biochemical Profile

Compared with the control group, the plasma ammonia level in the model group was significantly increased (*p* < 0.0001). Compared with the model group, AL0042 showed significant therapeutic effect (*p* < 0.001). AST, ALT in serum, markers of liver function, and injury appeared to be significantly increased in model animals compared with control animals (*p* < 0.0001), whereas the AL0042 treatment group counteracted these parameters (*p* < 0.05).

Systemic inflammation was observed because serum TNF-α levels increased in the model group compared to the control group (*p* < 0.001). Compared with the model group, AL0042 was shown to be protective against inflammation (*p* < 0.001).

SOD enzyme activity in serum was also evaluated to assay the antioxidant capacity of AL0042. Compared with the control group, SOD activity in the model group was significantly lowered (*p* < 0.01), but the AL0042 treatment (20 mg/kg) group significantly raised the levels (*p* < 0.05), suggesting a protective activity against oxidative damage (Table 6).

Blood corticosterone as the main glucocorticoid involved in the regulation of stress responses in rodents was also evaluated. The hormone increased in TAA-induced mice (*p* < 0.01), but it was significantly downregulated by AL0042 treatment (*p* < 0.05). These results are shown in Table 6.

### 3.5. Activity of AL0042 on Liver Morphology and Inflammation of TAA-Mice

**Figure 4:**
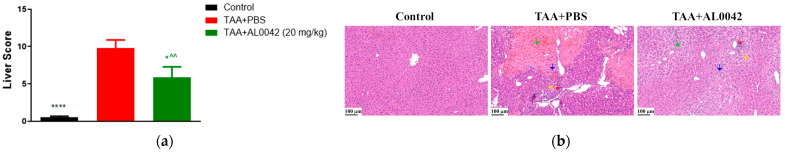
Activity of AL0042 on liver morphology and inflammation: (**a**) Liver score, with measurements performed in triplicate, and the error bars representing the standard error of the mean; (**b**) Hepatocyte morphological alteration. The samples were photographed under a light microscope using a 20× objective lens. An image of each slice was captured. **** *p* < 0.0001 versus TAA + PBS, * *p* < 0.05 versus TAA + PBS, ^^ *p* < 0.01 versus control. General score system for MHE staining: green arrow indicates congestion, red arrow indicates inflammation, blue arrow indicates necrosis, yellow arrow indicates vacuolization.

In TAA-treated mice, liver morphological evaluations, assessed based on Table 2, presented degenerated hepatocytes showing significant congestion, vacuolization, inflammation, and necrosis compared with the normal group’s livers (*p* < 0.0001) (Figure 4a). Interestingly, AL0042 treatment impeded hepatocyte morphological alteration induced by TAA in a significant manner (*p* < 0.05). Representative images are shown in Figure 4b.

### 3.6. Effect AL0042 on Brain Inflammation of TAA Mice

**Figure 5:**
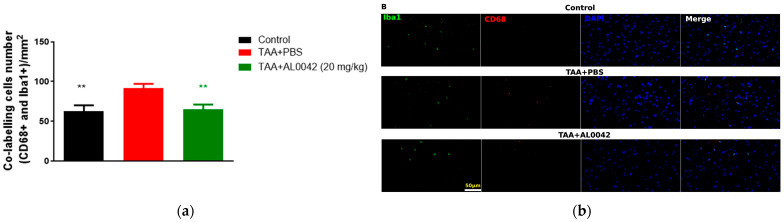
Brain inflammation markers: (**a**) IBA1/CD68 co-labelling cells number, with measurements performed in triplicate, and the error bars representing the standard error of the mean; ** *p* < 0.01 versus TAA + PBS were reported in green (TAA + AL0042) or black (control); (**b**) The samples were examined under a fluorescence microscope using the same magnification of image (40×). The quantification of cell numbers was performed by Image J.4. Three images of each slice were captured. IBA1/CD68 co-labelling cells numbers were counted by Image J.4, and the cells numbers were analyzed by normalizing per field, the area of which is 0.066 mm^2^; then, we averaged the data.

IBA1 to mark microglia in M1 and CD68 for activated microglia were used. The IBA1/CD68 co-labelling cells number indicated an activation ratio of microglia in sick mice compared to normal mice (*p* < 0.01), and there was a significant decrease in the 20 mg/kg AL0042 treatment group compared with the model group (*p* < 0.01), suggesting an anti-inflammatory effect of AL0042 also in the brain (Figure 5a,b).

## 4. Discussion

Our in vitro studies demonstrated that AL0042 spray-dried extract had interesting intestinal barrier integrity-protecting effects. These effects appeared to be only partly ascribed to hesperidin, thereby confirming the important role of the phytocomplex in determining the biological activity of the product. Indeed, an early pilot in vivo study on another murine model (not shown) using either isolated pectin or hesperidin revealed remarkably lower anti-inflammatory effects. Moreover, hesperidin was found to improve motor and cognitive ability in thioacetamide-induced hepatic encephalopathy in rats [36].

HC-based integral extracts of red orange by-products were found to consist of stable phytocomplexes with hesperidin adsorbed onto the surface of pectin [14]. Based on the prebiotic properties of pectin, which have been known for a long time [37], pectin could also give AL0042 prebiotic properties, which are currently under investigation. The mechanisms underlying the generation of citrus pectin-polyphenol conjugates were identified; for example, in the case of hydroxytyrosol, the adsorption onto the surface of pectin results in relatively weak non-covalent bonds, and the free radical method produces stronger covalent bonds [38]. It can be hypothesized that HC processes intensify both conjugation mechanisms: adsorption, due to the greatly enhanced mass transfer rate produced by the HC-induced turbulence and, likely more importantly, the formation of strong covalent bonds. The latter can be boosted due to the HC-based effective generation of hydroxyl radicals (•OH) [39,40], which would even avoid the need for ascorbic (or citric) acid as the reaction catalyst, leading to highly stable conjugates according to the process described by Rubio-Senent et al. [38].

The biological activity might be underestimated within our in vitro settings, since the production of butyrate would plausibly enhance the protective effects observed at the intestinal level in vivo. On the other hand, the anti-inflammatory activity appeared to be poor. This suggestion was also sustained by experiments conducted in undifferentiated Caco-2 cells, in which a bare inhibitory effect on NF-κB driven transcription (−14%) was observed after treatment with AL0042. Indeed, the experimental arrays conducted revealed no noteworthy anti-inflammatory activities associated with the extract. As well, a modest inhibitory effect was seen for a few genes, including CXCL-10, CXCL-11, and IL-16; INFγ is a known mediator of lymphocyte and monocyte activation; thus, downregulation of these genes could be related to the impairment of the INFγ pathway. Our experimental findings indicate that the extract is an antioxidant and rich in polyphenols, and flavonoids including hesperidin have been demonstrated to exert anti-inflammatory activities, according to the literature [18]. Of note, a relevant effect on liver inflammation, also playing a crucial role in MHE, was already suggested by previous studies (for a review on the topic, see [19]).

Hepatic diseases can lead to MHE that presents cognitive deficits [41]. By using the murine model induced by thioacetamide (TAA), it was shown that TAA was able to induce sever liver damage monitored with increased serum levels of hepatic enzymes and histopathological analysis of the liver. These hepatic modifications were associated with neuropsychiatry changes. TAA was already tested in different studies in rats and mice to analyze acute or chronic liver failure [42]. This study demonstrates the in vivo efficacy of the novel botanical compound AL0042 in targeting and modulating microglia, the primary immune cells in the brain. These cells can be activated by peripheral inflammatory cytokines in MHE patients [43]. The significant in vivo effects of orally administered AL0042 on both mouse behavior and the inflammation status of different tissues, such as the liver and brain, also points to an enhanced bioaccessibility of the phytocomplex compared to the pure hesperidin. This would confirm previous hypotheses that ascribed the enhanced bioaccessibility to the protection of hesperidin from gastric degradation and the enhanced water solubility, both due to hesperidin’s stable conjugation in the phytocomplex [44].

The MHE mouse model exhibited an increased density of activated microglial cells (CD68+Iba1+), along with higher serum levels of TNF-α and ammonia. These findings are consistent with observations reported in other models of liver injury [45,46]. Furthermore, microglia activation has been observed in different neurological disorders, such as Alzheimer’s disease [47], autism [48], and multiple sclerosis [49]. A hypothesis could therefore be advanced about further potential pharmacological activities of AL0042 in neurological diseases involving microglia activation, such as those reported for hesperidin [50]. However, the verification of this hypothesis, which is beyond the scope of the current study, is recommended as a subject of further research. The MHE murine model may be a suitable tool to highlight the possible efficacy of new products such as AL0042 to face this pathology. It was not possible to analyze microbiome profiles in this experimentation, and this may be a bias, although in vitro experiments suggested a beneficial impact on leaky gut symptoms. The mechanism underneath TEER recovery was not addressed; however, the literature already suggests the potential involvement of metabolic pathways including AMP-activated protein kinase (AMPK) [51]. Though, altogether, the in vivo behavioral data and ex vivo results obtained by the analysis of markers in peripheral and central tissues prompt us to further investigate the effectiveness of AL0042 in future clinical investigations on patients with cirrhosis and MHE. Future research could also provide further insights on the biological pathways leading to the observed in vivo effects.

The evidence provided also points to the unique role of HC, not only as a green, effective, efficient, and scalable extraction technique, but also due to its ability to generate a stable conjugated phytocomplex, without any additive other than water, endowed with enhanced biological functions. This supports the warrant for further investigation into the opportunity offered by the HC extraction method for a sustainable exploitation, up to the industrial production capacity, of the currently discarded or underutilized by-products of the red orange supply chain, which was already devised in a previous study [44].

In this study, the processes used to produce the extracts ALE1 and ALE2 were performed with the same HC regime, i.e., the same levels of the cavitation number in the reactor and pump impeller zones. Due to the sensitivity of the HC-driven generation rate of hydroxyl radicals (•OH) on the cavitation number [40], the hypothesized role of the same radicals in the production of the stable conjugated phytocomplexes, and the dependence of the cavitation number on the inlet pressure, thus on the consumed power, future studies should investigate the sensitivity of the results to the cavitation number.

Finally, only the extract ALE2 was used for the experiments; thus, the sensitivity of the results to the observed differences between ALE1 and ALE2, however small, are unknown, which represents another limitation of this study. As well, a higher degree of variability in the composition of AL0042 due to differences in the source material (red orange by-products) cannot be ruled out. In the perspective of industrialization, an evaluation is needed for both the standardization of the product obtained using variable source materials and a more in-depth characterization of its composition.

## 5. Conclusions

In conclusion, AL0042 showed promising protective activity at the intestinal level in vitro and a protective effect in a TAA-induced MHE in vivo model, showing positive effects on mouse behavior and on the inflammation status of the liver and brain, indicating potential therapeutic benefits and warranting further clinical investigation. Compelling evidence also ascribes a specific role to the HC-based extraction technique for the efficient production of a stable and effective phytocomplex, potentially paving the way for a sustainable, highly valuable exploitation of red orange by-products. The hesperidin contribution to the effect could be essential, although the effect of the whole phytocomplex seems to play a great role in the effects herein reported.

## Figures and Tables

**Table 1:** Analyzed quantities of the proximate and chemical composition of the spray-dried powder obtained from the aqueous extracts of ALE1 and ALE2.

Chemical Composition	Reference Method ^1^	References
Moisture	MP 2290 rev 6 2023 (ISO3727-1)	[22]
Proteins	MP 1457 rev 4 2022 (ISO14891:2002)	[23]
Total fats	MP 2598 rev 0 2022	[24]
Dietary fiber	MP 2135 rev 6 2021 (AOAC 991.43 1994)	[25]
Ash	MP 2271 rev 1 2022	[24]
Carbohydrates	MP 0297 rev 7 2021	[24]
Energy level	MP 0297 rev 7 2021	[24]
Organic acids	MP 0369 rev 4 2021	[24]
Sugars	MP 1114 rev 6 2016 (ISO22184)	[26]
Pectin	IFU 26/1996	[24]
Essential oils	AR 2019/120/ACAP.1	[24]
Specific volatiles	% GC area ^2^	[24]
Dietary fiber	MP 2443 rev 3 2022 (AOAC 2017.16)	[27]
Phenolic acids	MP 1337 rev 3 2014	[24]
Acidic composition	MP 2341 rev 1 2021 (ISO 16958)	[28]
Flavonoids	MI_197_2014_Rev3	[24]

^1^ Some analyses were carried out following internal methods without a correspondence with official methods. Where such correspondence exists, it has been indicated in brackets. The correspondence among internal and official methods was also described in a previous study [24]. ^2^ Proportion(%) of area in gas-chromatography (non-quantitative). The gas-chromatography analysis was performed coupled to a mass detector GC/MS (qualitative analysis) and GC/FID (semi-quantitative analysis) using a non-polar capillary column. The sample was extracted using a Clevenger SDE (Simultaneous Distillation and Extraction).

**Table 2:** Standard of Pathological Score.

Lesion	Lesion Characteristics	Score Values
Congestion	None	0
	Minimal	1
	Mild	2
	Moderate	3
	Severe	4
Vacuolization	None	0
	Minimal	1
	Mild	2
	Moderate	3
	Severe	4
Inflammation	None	0
	Minimal	1
	Mild	2
	Moderate	3
	Severe	4
Necrosis	None	0
	Minimal	1
	Mild	2
	Moderate	3
	Severe	4

**Table 3:** Component substances and PSD of the product, including AL0042, prepared from dry extracts of tests ALE1 and ALE2, and extraction yield. In brackets, the number of extraction tests.

Quantity	Level		Unit
	ALE1 (*n* = 6)	ALE2 (*n* = 6) (AL0042)	
Component substances			
Red orange byproducts	74.97 ± 0.13	74.86 ± 0.04	%
Maltodextrin	20.08 ± 0.11	20.10 ± 0.02	%
HPMC	4.95 ± 0.12	5.05 ± 0.03	%
PSD ^1^			
d10	2.42 ± 0.61	2.01 ± 0.30	μm
d50	5.58 ± 1.11	5.91 ± 0.93	μm
d90	10.65 ± 1.81	11.98 ± 1.85	μm
Span ^2^	1.49 ± 0.73	1.69 ± 0.63	
Spray drying recovery yield ^3^	86.97 ± 5.55	79.75 ± 3.62	%
Extraction yield ^4^	37.27 ± 1.62	37.93 ± 0.37	%

^1^ Particle size distribution. d10(50,90) = 10(50,90) % of the particles have sizes smaller than the respective indicated level. ^2^ $Span=\frac{d90-d10}{d50}$. ^3^ Spray drying recovery rate of the total dissolved solids (TDS) available in the aqueous extract, measured using the thermobalance. ^4^ Recovered active dry extract as a percentage of the dry raw material (red orange by-products).

**Table 4:** Proximate and chemical composition of the product, including AL0042, prepared from dry extracts of tests ALE1 and ALE2.

Chemical Composition	Quantity		Unit
	ALE1 (*n* = 6)	ALE2 (*n* = 6) (AL0042)	
Nutritional levels ^1^			
Moisture		10.36 ± 0.37	g/100 g
Proteins		3.98 ± 0.28	g/100 g
Total fats		0.74 ± 0.09	g/100 g
Dietary fiber		4.18 ± 0.67	g/100 g
Ash		2.55 ± 0.17	g/100 g
Carbohydrates		78.19 ± 0.84	g/100 g
Energy level		1438 ± 10	kJ/100 g
Organic acids			
Citric acid	1.50 ± 0.18	2.14 ± 0.26	g/100 g
Sugars			
Glucose	19.90 ± 1.50	15.60 ± 1.20	g/100 g
Fructose	20.60 ± 1.40	15.50 ± 1.00	g/100 g
Sucrose	8.04 ± 0.78	9.92 ± 0.96	g/100 g
Maltose	1.88 ± 0.28	1.59 ± 0.24	g/100 g
Total sugars	50.42 ± 2.21	42.61 ± 1.85	g/100 g
Pectin	1.78 ± 0.18	3.10 ± 0.31	g/100 g Gala eq ^2^
Essential oils ^3^	0.90 ± 0.03	1.10 ± 0.20	%*v*/*w*
Limonene	84.82 ± 0.03	95.76 ± 0.03	% GC aread
β-myrcene	2.18 ± 0.02	1.82 ± 0.02	% GC aread
α-pinene	0.82 ± 0.01	0.55 ± 0.01	% GC aread
Dietary fiber			
Soluble fiber	1.06 ± 0.25	1.84 ± 0.44	g/100 g
Total fiber	3.34 ± 0.60	9.54 ± 1.95	g/100 g
HMW fiber ^4^	2.28 ± 0.55	7.70 ± 1.90	g/100 g
Phenolic acids			
Chlorogenic acid	<0.01	0.07 ± 0.01	g/100 g
Caffeic acid	<0.01	0.02 ± 0.01	g/100 g
Ferulic acid	0.03 ± 0.01	0.02 ± 0.01	g/100 g
Acidic composition ^1^			
Saturated fatty acids		0.19 ± 0.03	g/100 g
Monounsaturated fatty acids		0.15 ± 0.02	g/100 g
Polyunsaturated fatty acids		0.32 ± 0.05	g/100 g
Flavonoids			
Hesperidin	1.71 ± 0.20	2.42 ± 0.28	g/100 g

^1^ Available only for ALE2. ^2^ Galacturonic acid equivalent. ^3^ Only the three most abundant aromatic volatiles are listed below. ^4^ HMW = high molecular weight.

**Table 5:** List and position of the 84 inflammatory genes in the heatmap presented in Figure 2c–e.

	01	02	03	04	05	06	07	08	09	10	11	12
A	AIMP1	BMP1	C5	CCL1	CCL11	CCL13	CCL15	CCL16	CCL17	CCL2	CCL20	CCL22
B	CCL23	CCL24	CCL26	CCL3	CCL4	CCL5	CCL7	CCL8	CCR1	CCR2	CCR3	CCR4
C	CCR5	CCR6	CCR8	CD40LG	CSF1	CSF2	CSF3	CX3CL1	CX3CR1	CXCL1	CXCL10	CXCL11
D	CXCL12	CXCL13	CXCL2	CXCL3	CXCL5	CXCL6	CXCL9	CXCR1	CXCR2	FASLG	IFNA2	INFG
E	IL10RA	IL10RB	IL13	IL15	IL16	IL17A1	IL17C	IL17F	IL1A	IL1B	IL1R1	IL1RN
F	IL21	IL27	IL3	IL33	IL5	IL15RA	IL7	CXCL8	IL9	IL9R	LTA	LTB
G	MIF	NAMPT	OSM	SPP1	TNF	TNFRSF11B	TNFSF10	TNFSF11	TNFSF13	TNFSF13B	TNFSF4	VEGFA

**Table 6:** Effect of AL0042 on plasma and serum parameters in TAA-induced mice. Data are mean ± SEM of all animals from different groups (*n* = 10 for each group). Superscripts show the significance of the differences: ^^ *p* < 0.01; ^^^ *p* < 0.001, versus control. * *p* < 0.05; ** *p* < 0.01; *** *p* < 0.001; **** *p* < 0.0001 versus TAA.

	Control	TAA	TAA + AL0042
Plasma ammonia			
NH_3_ (μmol/L)	31 ± 2.5 ****	87 ± 6.5	46.9 ± 2.0 ^^^***
Serum parameters			
ALT (U/L)	39.6 ± 5.0 ****	116.8 ± 13.4	72.3 ± 6.3 ^^^*
AST (U/L)	88.0 ± 3.6 ****	200.9 ± 16.6	140 ± 13 ^^^*
TNF-α (ng/mL)	1.0 ± 0.2 ***	46.8 ± 11.5	1.0 ± 0.3 ^^^
SOD activity (% inhibition rate)	79.3 ± 0.5 **	72.9 ± 2.0	78.2 ± 1.3 *
Corticosterone (ng/mL)	52.8 ± 6.0 **	243 ± 75	87.8 ± 9.6 *^^

## Data Availability

The data that support the findings of this study are available from the corresponding authors upon reasonable request.

## Abbreviations

The following abbreviations are used in this manuscript:

MHE	Minimal hepatic encephalopathy
AL0042	New phytocomplex extracted from red orange by-products
TAA	Thioacetamide
ACLF	Acute and chronic liver failure
AST	Aspartate aminotransferase
ALT	Alanine aminotransferase
SOD	Superoxide dismutase
HC	Hydrodynamic cavitation
DE	Degree of esterification
ALE1	First HC extraction run
ALE2	Second HC extraction run
σ	Cavitation number
PSD	Particle size distribution
HPMC	Hydroxypropyl methylcellulose
ORAC	Oxygen radical absorbance capacity
AAPH	2,2′-azobis(2-methylpropionamidine) dihydrochloride
AUC	Area under the curve
MTT	3-(4,5-dimethyl-2-thiazolyl)-2,5-diphenyl-2H-tetrazolium bromide
TEER	Transepithelial electrical resistance
FC	Fold Change
AAALAC	Applicable assessment and accreditation of laboratory animal care
IACUC	Institutional Animal Care and Use Committee
TNF-α	Tumor necrosis factor-alpha
PBF	Phosphate-buffered saline
GFAP	Glial fibrillary acid protein
CD68	Macrophage/phagocytic activation
IBA-1	Ionized calcium-binding adapter molecule-1
TDS	Total dissolved solids
TPC	Total phenolic content
